# 

**Published:** 1847-10-01

**Authors:** 


### CONTENTS

OF THE

### MEDICO-CHIRURGICAL REVIEW,

### OCTOBER 1, 1847.

### REVIEWS.

I.

The Human Brain; its Structure, Physiology, and Diseases. By SAMUEL SOLLY, F.R.S., &c. .. .. .. .. .. .. .. .. .. .. .. .. .. .. .. 285

1. Comparative Anatomy of the Encephalon .. .. .. .. .. .. .. 287

2. True Mode of Studying the Brain .. .. .. .. .. .. .. .. 289

3. Relations of the Cerebellum and Spinal Cord .. .. .. .. .. 290

4. Anatomical Conclusions .. .. .. .. .. .. .. .. .. .. .. 293

5. Seat of Sensation and Volition .. .. .. .. .. .. .. .. 295

6. On Anæmic Coma .. .. .. .. .. .. .. .. .. .. .. .. 297

7. Principles of treating Insanity.. .. .. .. .. .. .. .. .. 299

8. Pathology of Epilepsy .. .. .. .. .. .. .. .. .. .. 300

II.

Ricerche ed Esperienze sul Sangue Umano. Del Dottore GIO POLLI .. .. .. 303
1. Researches and Experiments upon the Formation of the Buffy Coat of the Blood, &c. .. .. .. .. .. .. .. .. .. .. .. .. .. 303
2. On a New Criterion for the Regulation of Blood-letting .. .. .. 310
3. On the Condition of the Fibrine of the Blood in Inflammation .. .. 314
4. On the Effects of the Abstraction of Blood upon the Human Organism 319
5. Modifications of the Blood by Bleeding.. .. .. .. .. .. .. .. 321
6. Practical Applications .. .. .. .. .. .. .. .. .. .. .. 323

III.

The Physiological Anatomy and Physiology of Man. Part the Third. By ROBERT BENTLEY TODD, M.D. F.R.S. &c. and WILLIAM BOWMAN, F.R.S. .. .. .. 326

1. On Sensation and Perception.. .. .. .. .. .. .. .. .. .. .. 327

2. Structure of the Cornea, Iris, &c... .. .. .. .. .. .. .. .. 328

3. Structure of the Retina, of the Cochlea, &c... .. .. .. .. .. .. 330

IV.

I. The American Journal of Insanity .. .. .. .. .. .. .. .. .. ..
II. Fourth Annual Report of the Managers of the State Lunatic Asylum of New York .. .. .. .. .. .. .. .. .. .. .. .. .. .. ..
III. Remarques sur quelques Etablissemens d'Aliénés de la Belgique, de la Hollande, et de l'Angleterre. Par M. BRIERRE DE BOISMONT .. .. } 333
1. Shakespeare's Delineations of Insanity .. .. .. .. .. .. .. .. 333
2. Medical Treatment of Insanity at the New York State Lunatic Asylum 339
3. Frequency of Pulse of the Insane—Size and Shape of the Head.. .. 340
4. Weight of the Insane—Hereditary Predisposition.. .. .. .. .. .. 341
5. Suicidal Form of Insanity—Homicidal Insanity .. .. .. .. .. .. 342
6. Plea of Insanity .. .. .. .. .. .. .. .. .. .. .. .. .. 344
7. On Establishments for the Insane in Belgium, Holland and England.. 346
8. Objections to the non-restraint System.. .. .. .. .. .. .. .. 349

V.

I. On Pulmonary Consumption, and on Bronchial and Laryngeal Disease, &c. By Sir CHARLES SCUDAMORE, M.D., F.R.S. &c. .. .. .. .. .. ..
II. Cold and Consumption. By HENRY C. DESHON, M.D. &c. .. .. .. } 351

1. Treatment with the Inhalation of Iodine .. .. .. .. .. .. .. 353
2. Great Increase of the Animal Heat in Phthisis .. .. .. .. .. 355
3. Increase of the Carbonic Acid during Respiration .. .. .. .. 357
4. Effects of inhaling the Vapour of Water .. .. .. .. .. .. .. 359

VI.

A System of Surgery. By J. M. Chelius.. .. .. .. .. .. .. .. .. .. 360
1. Elementary Proceedings of Surgical Operations .. .. .. .. .. .. 361
2. Treatment after Operations .. .. .. .. .. .. .. .. .. .. .. 363
3. General Treatment of Lithotomy Patients .. .. .. .. .. .. .. 364
4. Comparison of Lithotomy and Lithotrity .. .. .. .. .. .. .. 366
5. Radical Cure of Hydrocele .. .. .. .. .. .. .. .. .. .. .. 369
6. Question of removing a Scirrhous Breast .. .. .. .. .. .. .. 370
7. Application of Blisters .. .. .. .. .. .. .. .. .. .. .. .. 371
8. Use of the Saw in Amputation .. .. .. .. .. .. .. .. .. .. 372
9. Results of Amputation—Circumcision in Phymosis .. .. .. .. 373
10. Reduction of Paraphymosis .. .. .. .. .. .. .. .. .. .. .. 374
11. Production and Treatment of Curvatures .. .. .. .. .. .. .. 375

VII.

On the Causes and Treatment of Abortion and Sterility. By James Whitehead, F.R.C.S. &c.. .. .. .. .. .. .. .. .. .. .. .. .. .. .. .. .. 377

1. Physical Properties of the Menstrual Fluid .. .. .. .. .. .. 378

2. Spurious Menstruation .. .. .. .. .. .. .. .. .. .. .. .. 380

3. Conditions influencing Menstruation .. .. .. .. .. .. .. .. 382

4. Dysmenorrhœa—Menorrhagia .. .. .. .. .. .. .. .. .. .. 384

5. Last Menstrual Crisis .. .. .. .. .. .. .. .. .. .. .. .. 386

6. Uterine Diseases liable to occur at the Crisis .. .. .. .. .. .. 387

7. Signs of Pregnancy .. .. .. .. .. .. .. .. .. .. .. .. .. 390

8. Menstruation during Pregnancy .. .. .. .. .. .. .. .. .. .. 393

9. Abortion; its Statistics and Causes .. .. .. .. .. .. .. .. 395

10. Leucorrhœa—Endo-uteritis .. .. .. .. .. .. .. .. .. .. .. 397

11. Gonorrhœal Inflammation of the Uterus .. .. .. .. .. .. .. 398

12. Sterility—Illustrative Cases .. .. .. .. .. .. .. .. .. .. 400

VIII.

I. Researches on the Chemistry of the Food. By Justus Liebig .. .. .. } 405

II. The Chemistry of Vegetable and Animal Physiology. By Dr. G. J. Mulder }

1. Insufficiency of Ultimate Analysis.. .. .. .. .. .. .. .. .. .. 407

2. Composition of the Juices of Flesh.. .. .. .. .. .. .. .. .. .. 409

3. Rationale of Cooking Processes .. .. .. .. .. .. .. .. .. .. 410

4. Chemico-Histology.. .. .. .. .. .. .. .. .. .. .. .. .. .. 413

IX.

Further Report of the Commissioners in Lunacy to the Lord Chancellor .. .. 415
1. Improved Condition of the Insane.. .. .. .. .. .. .. .. .. .. 417
2. Imprisonment of a Lunatic for Debt—Lincoln Asylum .. .. .. 419
3. Treatment of Insanity in the English Asylums .. ...... .. .. 421

X.

Théorie Expérimentale de la Formation des Os. Par P. Flourens .. .. .. 426

1. Powers of the Periosteum in forming Bone .. .. .. .. .. .. .. 427

2. Powers of the Endosteum.. .. .. .. .. .. .. .. .. .. .. .. .. 429

XI.

Report on the Climate and principal Diseases of the African Station. By Alexander Bryson, M.D. &c. .. .. .. .. .. .. .. .. .. .. .. .. .. .. .. 431
1. Topography of Sierra Leone .. .. .. .. .. .. .. .. .. .. .. 432
2. Yellow Fever on board the "Bann," &c. in 1823 .. .. .. .. 434

3. Yellow Fever on board the "Eden" in 1829 .. .. .. .. .. .. 436
4. Yellow Fever on board the "Sybille" in 1829 .. .. .. .. .. 439
5. Medical History of Fernando Po .. .. .. .. .. .. .. .. .. .. 440
6. Yellow Fever on board several Ships in 1838.. .. .. .. .. .. 442
7. Epidemic Diarrhœa a precursor of Yellow Fever .. .. .. .. .. 445
8. Yellow Fever on board the "Eclair" .. .. .. .. .. .. .. .. 446
9. Dr. Bryson's Views respecting the Nature of Yellow Fever.. .. .. 449
10. Remarks on the Treatment of Yellow Fever.. .. .. .. .. .. .. 451

XII.

A Treatise on the Physical Cause of the Death of Christ, and its relation to the Principles and Practice of Christianity. By WILLIAM STROUD, M.D. .. .. 453

1. Influence of strong Emotions on the Heart, &c. .. .. .. .. .. 455

2. Instances of Sanguineous Perspiration .. .. .. .. .. .. .. 457

3. Rupture of the Heart from intense Emotion.. .. .. .. .. .. .. 458

4. Separation of Effused Blood into Clot and Serum .. .. .. .. 461

5. Effusion of Blood into the Pericardium.. .. .. .. .. .. .. .. 463

6. Punishment of Crucifixion described .. .. .. .. .. .. .. .. 465

7. Crucifixion a lingering Mode of Death .. .. .. .. .. .. .. .. 466

8. Suddenness of our Saviour's dissolution .. .. .. .. .. .. .. 468

9. Physical Cause of our Saviour's Death explained .. .. .. .. 471

XIII.

Memoir sur la Rougeole des Adultes. Par le Docteur MICHEL LEVY .. .. .. 477

1. Form and Progress of the Eruption .. .. .. .. .. .. .. .. 477

2. Concomitants of the Disease .. .. .. .. .. .. .. .. .. .. .. 478

3. Complications of the Disease.. .. .. .. .. .. .. .. .. .. .. 479

4. Consecutive Maladies .. .. .. .. .. .. .. .. .. .. .. .. 480

5. Antagonism—Mortality .. .. .. .. .. .. .. .. .. .. .. .. 481

6. Treatment.. .. .. .. .. .. .. .. .. .. .. .. .. .. .. .. 482

XIV.

I. Copy of Reports and Communications on M. Ledoyen's Disinfecting Fluid }
II. Copy of Reports on Sir William Burnett's Disinfecting Fluid .. .. .. } 483
1. Solutions of Nitrate of Lead and Chloride of Zinc .. .. .. .. .. 485
2. Their Antibromic and Antiseptic Properties .. .. .. .. .. .. .. 487
3. Have they any power over Infectious Miasms .. .. .. .. .. .. 489
4. Results of Experiments in Dublin.. .. .. .. .. .. .. .. .. .. 492

XV.

Traité Theoretique et Pratique d'Auscultation Obstetricale. Par J. H. A. DEPAUL, D.M., &c. .. .. .. .. .. .. .. .. .. .. .. .. .. .. .. .. .. .. 495

1. The Uterine Souffle or Blowing Sound .. .. .. .. .. .. .. .. 497

2. The Cardiac Pulsation of the Fœtus .. .. .. .. .. .. .. .. 500

XVI.

On the System of the Great Sympathetic Nerve. By C. RADCLYFFE HALL, M.D. 506

XVII.

Observations on the Treatment of Lateral Curvature of the Spine, &c. &c. By E. F. LONSDALE, F.R.C.S.E. &c. .. .. .. .. .. .. .. .. .. .. 510

XVIII.

Cholera, Dysentery, and Fever, pathologically and practically considered. By CHARLES SEARLE, M.D. &c. .. .. .. .. .. .. .. .. .. .. .. .. 515

XIX.

Remarks on Vivisection, and on certain Allegations as to its Utility in the Study and Application of Physiology. By GEORGE MACILWAIN, F.R.C.S., &c. .. 518

XX.

Report on the Archetype and Homologies of the Vertebrate Skeleton. By Professor OWEN, F.R.S. &c... .. .. .. .. .. .. .. .. .. .. .. .. .. .. 520

XXI.

The Microscopic Anatomy of the Human Body in Health and Disease. By ARTHUR HILL HASSALL .. .. .. .. .. .. .. .. .. .. .. .. .. .. 522

XXII.

The Preservation of Infants in Delivery. By RICHARD KING, M.D., M.R.C.S. .. 522

### PERISCOPE.

### Selections from the Foreign Periodicals.

1. On the Frequency of the Pulse and Respiration in the Aged. By C. W. Pennock, M.D. .. .. .. .. .. .. .. .. .. .. .. .. .. .. .. .. 523
2. On Scorbutus as observed in the Salpêtriére in 1847, and on the Composition of the Blood in this Disease. By Dr. Fauvel .. .. .. .. .. .. .. 525
    1. Andral on the Blood in Scorbutus.. .. .. .. .. .. .. .. .. .. 527
    2. Analogy between Scorbutus and Typhus .. .. .. .. .. .. .. 529
3. Influence of Electricity in the production of Diseases. By M. Pallas .. .. 531
4. On the Diagnostic Signs furnished by Inspection of the Vulva and the Entrance of the Vagina. By M. Huguier .. .. .. .. .. .. .. .. .. .. .. .. 531
5. On Stricture of the Intestine in Hernia. By Louis Chapel .. .. .. .. .. 533
6. Researches on the Normal and Anormal Capacity of the Cavities of the Heart. By Dr. Beau .. .. .. .. .. .. .. .. .. .. .. .. .. .. .. .. .. .. 534
7. Eschars over the Sacrum .. .. .. .. .. .. .. .. .. .. .. .. .. .. 537
8. Painful Crepitation of the Tendons. By M. Velpeau .. .. .. .. .. .. 537
9. M. Royer-Collard on the Statistics of Insanity .. .. .. .. .. .. .. .. 538
10. M. Civiale, on the Statistics of Lithotrity and Lithotomy .. .. .. .. 539
11. On the Induction of Cystitis and Albuminuria by Blisters .. .. .. .. 540
12. On Abscess of the Breast. By M. Velpeau .. .. .. .. .. .. .. .. .. 543
13. Andral on the Intestinal Secretions in Cholera .. .. .. .. .. .. .. 544
14. Induction of Premature Labour in Miscarriages.. .. .. .. .. .. .. .. 545
15. Purulent Ophthalmia of New-born Infants .. .. .. .. .. .. .. .. 547
16. Serres on the Treatment of Typhoid Fever by the Black Sulphuret of Mercury 549
17. The Medical Profession in Paris .. .. .. .. .. .. .. .. .. .. .. .. .. 551
18. Observations on the Materia Medica .. .. .. .. .. .. .. .. .. .. .. 552
    1. Mialhe on Alkaline Medicines .. .. .. .. .. .. .. .. .. .. 552
    2. Alum Gargles .. .. .. .. .. .. .. .. .. .. .. .. .. .. .. .. 554
    3. Astringent Collyria .. .. .. .. .. .. .. .. .. .. .. .. .. .. 554
    4. Sedative Cataplasm in Puerperal Arthritis .. .. .. .. .. .. .. 555
    5. Disguise of the Bitterness of Medicines by means of Coffee .. .. 555
    6. The Employment of Tonics in Acute Disease .. .. .. .. .. .. 556
19. Cazenave on Papular Diseases of the Skin .. .. .. .. .. .. .. .. .. 557
20. Prolonged Compression in Hiccough .. .. .. .. .. .. .. .. .. .. .. 558
21. Account of a Physical Sign of Pneumonia of the Apex of the Lung. By Wm. Boling, M.D... .. .. .. .. .. .. .. .. .. .. .. .. .. .. .. .. 559
22. New Modes of effecting Union of Wounds .. .. .. .. .. .. .. .. .. 559
23. Chronic Cutaneous Eruptions .. .. .. .. .. .. .. .. .. .. .. .. .. 559
Address to the Reader .. .. .. .. .. .. .. .. .. .. .. .. .. .. .. .. 560
Bibliographical Record .. .. .. .. .. .. .. .. .. .. .. .. .. .. .. .. 561

